# Pro-Resolving Factors Released by Macrophages After Efferocytosis Promote Mucosal Wound Healing in Inflammatory Bowel Disease

**DOI:** 10.3389/fimmu.2021.754475

**Published:** 2021-12-22

**Authors:** Omayra Martin-Rodriguez, Thierry Gauthier, Francis Bonnefoy, Mélanie Couturier, Anna Daoui, Cécile Chagué, Séverine Valmary-Degano, Claire Gay, Philippe Saas, Sylvain Perruche

**Affiliations:** ^1^ Univ. Bourgogne Franche-Comté, INSERM, EFS BFC, UMR1098 RIGHT, Interactions Hôte-Greffon-Tumeur/Ingénierie Cellulaire et Génique, Fédération Hospitalo-Universitaire INCREASE, LabEx LipSTIC, Besançon, France; ^2^ MED’INN’Pharma, Besançon, France; ^3^ Department of Pathology, University Hospital of Besançon, Besançon, France; ^4^ Department of Gastroenterology, University Hospital of Besançon, Besançon, France

**Keywords:** efferocytosis, resolution of inflammation, macrophages, fibroblasts, colitis, wound healing

## Abstract

Nonresolving inflammation is a critical driver of several chronic inflammatory diseases, including inflammatory bowel diseases (IBD). This unresolved inflammation may result from the persistence of an initiating stimulus or from the alteration of the resolution phase of inflammation. Elimination of apoptotic cells by macrophages (a process called efferocytosis) is a critical step in the resolution phase of inflammation. Efferocytosis participates in macrophage reprogramming and favors the release of numerous pro-resolving factors. These pro-resolving factors exert therapeutic effects in experimental autoimmune arthritis. Here, we propose to evaluate the efficacy of pro-resolving factors produced by macrophages after efferocytosis, a secretome called SuperMApo, in two IBD models, namely dextran sodium sulfate (DSS)-induced and T cell transfer-induced colitis. Reintroducing these pro-resolving factors was sufficient to decrease clinical, endoscopic and histological colitis scores in ongoing naive T cell-transfer-induced colitis and in DSS-induced colitis. Mouse primary fibroblasts isolated from the colon demonstrated enhanced healing properties in the presence of SuperMApo, as attested by their increased migratory, proliferative and contractive properties. This was confirmed by the use of human fibroblasts isolated from patients with IBD. Exposure of an intestinal epithelial cell (IEC) line to these pro-resolving factors increased their proliferative properties and IEC acquired the capacity to capture apoptotic cells. The improvement of wound healing properties induced by SuperMApo was confirmed *in vivo* in a biopsy forceps-wound colonic mucosa model. Further *in vivo* analysis in naive T cell transfer-induced colitis model demonstrated an improvement of intestinal barrier permeability after administration of SuperMApo, an intestinal cell proliferation and an increase of α-SMA expression by fibroblasts, as well as a reduction of the transcript coding for fibronectin (*Fn1*). Finally, we identified TGF-β, IGF-I and VEGF among SuperMApo as necessary to favor mucosal healing and confirmed their role both *in vitro* (using neutralizing antibodies) and *in vivo* by depleting these factors from efferocytic macrophage secretome using antibody-coated microbeads. These growth factors only explained some of the beneficial effects induced by factors released by efferocytic macrophages. Overall, the administration of pro-resolving factors released by efferocytic macrophages limits intestinal inflammation and enhance tissue repair, which represents an innovative treatment of IBD.

## 1 Introduction

Inflammatory bowel diseases (IBD, including Crohn’s disease [CD] and ulcerative colitis [UC]) consist in a family of chronic idiopathic relapsing diseases characterized by a dysregulated response of the innate and adaptive immune system. This dysregulated response leads to the damage of the intestine and of the colonic mucosa (1). Today, the most commonly available treatments for IBD are based on immunosuppressive agents that target the deleterious effector arm of the pathogenic immune response (2). However, these current approaches are not entirely effective and not specific to pathogenic immune cells triggering IBD. They are responsible for multiple adverse effects, including an increased susceptibility to infections. In most cases, surgical resection remains the ultimate therapeutic alternative. Despite significant improvements in the management of IBD patients brought by the development of innovative drugs [such as biologic agents like anti-TNF therapy (3, 4)], new therapeutic strategies are needed.

Non-immune components of the intestinal mucosa are also involved in the pathogenesis of IBD (5). This is illustrated by intestinal epithelial cell (IEC) damage which participates in IBD pathogenesis (6). Indeed, mucosal healing is emerging as a critical endpoint in clinical trials and practice (2, 7). Under normal homeostatic conditions, once neutrophils have controlled and eliminated the mucosal insult, they undergo apoptosis at the site of inflammation (8), and then attract blood-derived monocytes through “find-me” signals (9). Together with tissue-resident phagocytes, monocyte-derived macrophages eliminate apoptotic cells through a process called efferocytosis (10). Along with a pro-inflammatory to pro-resolving lipid shift (11), efferocytosis triggers the resolution of inflammation, leading to restoration of homeostasis and regeneration of a functional tissue (12). Factors produced by efferocytic macrophages promote wound repair (13). This repair involves the migration, proliferation and differentiation of different cell types, including macrophages, fibroblasts, as well as IEC in IBD (2). The secretome of macrophages triggered by apoptotic cell removal (thereafter called SuperMApo) demonstrates its ability to terminate chronic inflammation and restore tissue homeostasis in several inflammatory situations, particularly in ongoing arthritis (14). SuperMApo contains several factors, including chemokines (CCL5, CXCL2, and CCL22) and cytokines (IL-1RA, IL-10, and TGF-β) (14). In the treatment of collagen-induced arthritis with SuperMApo, TGF-β demonstrated a critical role in association with co-factors (14). In that context, SuperMApo induced auto-antigen-specific regulatory T cells through the reprogramming of antigen-presenting cells, in particular macrophages (14).

During intestinal inflammation, the differentiation of monocytes into mature intestinal macrophages is disrupted; this delays efferocytosis and aborts the resolution phase. Neutrophils accumulate in the *lamina propria* (15), resist transiently to apoptosis (16) and generate a highly inflammatory microenvironment contributing to tissue destruction (17). This favors a pro-inflammatory macrophage profile; these activated macrophages release inflammatory mediators (18, 19) preventing neutrophil apoptosis and delaying the pro-inflammatory to pro-resolving lipid shift (20, 21). This creates a pro-inflammatory loop.

Triggering an efficient resolution of inflammation in IBD seems an innovative approach to limit inflammation and favor tissue healing (22, 23). Apoptotic cell-based approaches have been successfully evaluated to trigger resolution in two experimental models of IBD, triggering resolution through the efficient elimination of apoptotic cells by phagocytes and the consequent release of pro-resolutive factors (24, 25). We therefore addressed whether the administration of pro-resolving factors released by macrophages after efferocytosis would be able to restore intestinal mucosa homeostasis. Here, we provide evidence that reintroducing these pro-resolving factors enhances tissue repair as the colon mucosa ameliorations observed in the DSS-induced chronic colitis model, showing similarities with UC (notably the presence of crypt abscesses), and in the T cell transfer-induced colitis showing similarities with CD (notably endoscopic and histological deep ulcerations and inflammatory cell infiltration), thanks to video-endoscopy. The same pro-healing properties of SuperMApo pro-resolutive drug were also observed in absence of systemic inflammation using a model of forceps-induced intestinal wound. Pro-resolving factors modulate local fibroblasts and IEC, which then acquire pro-resolving properties. Our findings reveal that pro-resolving factors released by macrophages after efferocytosis represent important modulators of mucosal inflammation and healing. This represents a new therapeutic approach for the treatment of IBD.

## 2 Material and Methods

### 2.1 Mice

Four to eight-week-old C57BL/6 female mice were obtained from Charles River Laboratories (L’Arbresle, France) and kept in quarantine for at least one week before any experimental procedure. Rag2-deficient (Rag-2^–/–^) C57BL/6 mice were bred in our specific pathogen-free rodent facility. Mice were housed in this facility (#D25-056-7) in standard plastic cages with cellulose bedding in environmentally controlled and germ-free rooms at 22°C, and kept under a 12-hour light/dark cycle. Pellet food and sterile tap water (Hydropack; Plexx) were available *ad libitum*. Experimentation (#02831) was approved by the local ethic committee (#58) and the French Ministry of Higher Education and Research (*Ministère de l’Enseignement Supérieur, de la Recherche et l’Innovation*) and was conducted in accordance with the European Union Directive 2010/63.

### 2.2 *In Vivo* Intestinal Mucosal Injury Procedure

An intestinal mucosal wound was performed as previously described (26). To wound the C57BL/6 mouse colon, a biopsy forceps was inserted into the working channel of the endoscope (superslim 8.4 Fr, 2.8 mm, flexible video ureteroscope [URF V2; Olympus]) and introduced into the colon of a 2% isoflurane-anaesthetized 6 to 8-week-old mice. Under visual control, a small piece of mucosa was carefully removed. SuperMApo or vehicle (X-vivo) was administrated i.p. the day of the injury and 48 hrs later (1 mL per mouse/injection). The process of wound healing was subsequently monitored by daily endoscopies.

### 2.3 Naive T Cell Transfer-Induced Colitis

Naive CD25^−^CD45RB^high^CD4^+^ T cells were sorted by flow cytometry (cell sorter SH800Z, SONY) from magnetically enriched CD4^+^ spleen and lymph node cell suspensions (EasySep Mouse CD4^+^ T Cell Isolation Kit; StemCell Technology) to a >95% purity. According to the described model (27), 5.10e5 naive T cells sorted from C57BL/6 were injected into the tail vein of 6 to 8-week-old Rag-2^–/–^ C57BL/6 mice to induce colitis. Mice were monitored daily for clinical assessment of colitis and twice a week by video-endoscopy to appreciate colon mucosa. SuperMApo or vehicle (X-vivo) was administrated i.p. at day 10, after T cell transfer, and 48 hrs later (1 mL/mouse/injection). At that time (10 days post-T cell transfer) mice experimented a 6 to 7 MEICS score, out of 15. In additional *in vivo* experiments, TGF-β, IGF-I and VEGF recombinant growth factors (R&D systems), or PBS, were administrated i.p. at day 10, after T cell transfer, and 48 hrs later (1 mL per mouse/injection; TGF-β: 700 pg/mL, IGF-I: 1050 pg/mL and VEGF 350 pg/mL).

### 2.4 Dextran Sulfate Sodium (DSS)-Induced Colitis

Chronic DSS colitis was induced in 6 to 8-week-old C57BL/6 mice with the addition of 3% of DSS (MP Biomedicals LLC) in drinking water for seven consecutive days. Then, regular water was given for the next 10 days, and this cycle was repeated twice. Control mice were given with tap water only. Mice were monitored daily for clinical assessment of colitis and twice a week by video-endoscopy to appreciate their colon mucosa. SuperMApo or vehicle (X-vivo) were given i.p. the day of the first DSS cycle initiation, and 48 hrs later (1 mL/mouse).

### 2.5 Mice Monitoring and Video-Endoscopy

The mice were monitored daily for clinical assessment of colitis according to a total clinical score of 0 to 9 assigned to each animal and composed of the sum of the body weight loss (0 to 5% of weight loss = 0; 5 to 10% = 1; 10 to 15% = 2; 15 to 20% = 3; >20% = 4), the feces appearance (normal = 0; diarrhea = 1; bloody stools = 2), and the general behavior (normal = 0; mild alteration = 1; piloerection and motor activity altered = 2; severe alteration = 3). The mice were monitored twice a week by video-endoscopy under 2% isoflurane-induced anesthesia, without bowel preparation nor starving. Video-endoscopy was performed using a superslim 8.4 Fr (2.8 mm) flexible video ureteroscope (URF V2; Olympus), connected to OTV-S190 and CLV-S190 visera elite monitor system (Olympus), with a narrow band imaging system enhancing visibility of vascular structures. Endoscopic assessment of mucosal inflammation was reported with the murine endoscopic index of clinical severity (MEICS) score (28), composed of five parameters rated from 0 to 3, including: thickening of the colon, changes in the vascular pattern, fibrin deposition, granularity of the mucosal surface, and stool consistency.

### 2.6 Intestinal Permeability Evaluation

The mice were submitted to gavage with fluorescein isothiocynate (FITC)-dextran (MW 4000; Sigma-Aldrich) at 80 mg/kg of body weight, five hrs prior to blood collection through retro orbital puncture. Fluorescence intensity in the serum was measured at 520 nm (Perkin Elmer). FITC-dextran concentrations were determined from a standard curve generated by a serial dilution of FITC-dextran. Quantification of regenerating islet derived protein 3-γ (REG-3γ) concentrations in plasma was determined by ELISA (Cloud clone corporation) according to the manufacturer’s instructions.

### 2.7 Generation of pro-Resolving Factors Released by Efferocytic Macrophages

Pro-resolving factors from mouse or human pro-resolving macrophages (called SuperMApo) were produced as described previously (14). Briefly, SuperMApo consisted in the supernatant of a 48-hr culture of apoptotic thymocytes (rendered apoptotic by a 35 Gy-irradiation) with thioglycolate-elicited peritoneal macrophages, in X-vivo culture medium to a 5 to 1 ratio, respectively (14). Human SuperMApo consisted in the 48-hr culture supernatant of M-CSF blood monocyte-derived macrophages cultured with apoptotic autologous peripheral blood mononuclear cells (rendered apoptotic by a 35 Gy-irradiation) to a 5 to 1 ratio in MEM medium, respectively (14). Supernatants were collected, centrifuged to eliminate debris, filtered through a 0.22 µm filter and frozen or lyophilized and frozen for conservation. SuperMApo was used directly in culture experiments (see *Functional Assays*) or injected i.p. for *in vivo* experiments (1 ml/mouse repeated 48 hrs later, in total 2 ml per mouse). In some *in vitro* experiments, VEGF (AF493NA), TGF-β (MAB240) and IGF-I (AF791) neutralizing antibodies or control anti-goat IgG (AB-108-C) or anti-mouse IgG1 antibodies (MAB002) (all from R&D systems), were used in addition to SuperMApo. In some *in vivo* experiments, growth factors were immuno-magnetically depleted from SuperMApo. Briefly, 10 µg/ml of antibodies directed against VEGF and IGF-I (the same clones than in blocking experiments) as well as 50 µg/ml of anti-TGF-β antibody (2G7 clone; provided by Prof. L. Chatenoud) were incubated for one hour with SuperMApo at room temperature. Then, goat anti-mouse IgG (Polysciences Europe GMBH) or rabbit anti-goat IgG (Rockland) coated magnetic beads were incubated separately with a pre-formed antigen (Ag)-antibody (Ab) complex for 20 minutes or one hour respectively, at room temperature. The Ag-Ab-bead complexes were then captured using a magnetic separator. Protein levels of FGF, EGF, TGF-β, IGF-I and VEGF in SuperMApo were determined by ELISA (all from R&D systems), according to the manufacturer’s protocols.

### 2.8 Histological Analysis and Immunofluorescence

Immediately after sacrificing the mice, tissue samples from the proximal, median and distal parts of the colon were collected and fixed overnight in 5% formalin. Paraffin inclusions were performed on the macroscopic injured area. Then, 4 µm sections were stained with hematoxylin-eosin-saffron (HES). A histological evaluation was performed by an experimented pathologist blinded to the nature of the mice being examined, using a five-degree severity score based on inflammatory cell infiltration, erosions and ulcerations, epithelial hyperplasia and mucin depletion, as previously described (29). Cytofix/Cytoperm buffer (eBioscience) was applied for the detection of nuclear staining. The primary antibodies used for immunostaining of mouse tissue and primary fibroblasts were: rabbit anti-mouse Ki-67 (clone SP6; Abcam), rabbit anti-human α-smooth muscle actin (α-SMA) (polyclonal; Abcam) and Alexa 488-conjugated rabbit anti-vimentin (Clone D21H3; Cell signaling). The secondary antibody was Alexa Fluor 555 goat anti-rabbit IgG (H+L) (Life Technologies). The nuclei were counterstained with DAPI (Sigma-Aldrich). Fluorescence analysis was performed with Olympus IX81 and Fluoview FV1000 software (Olympus). For quantitative analyses, at least four to five representative high-power fields (HPF, 40X) per section were evaluated in a blinded manner.

### 2.9 RNA Isolation and PCR Analysis

Total RNA were extracted from tissues or cells using the RNeasy mini kit (Qiagen) and 1 to 2 µg of RNA was reversed-transcribed into cDNA using the high capacity RNA to cDNA kit (Applied Biosystems). Then, cDNA were quantified by real time RT-PCR using the power SYBR green PCR master mix (Applied Biosystems) using *Col1a1* (Mm0080 1666_m1), *Col3a1* (Mm0125 4476_m1), *Fn1* (Mm0125 6744_m1) and *GAPDH* primers (from Applied Biosystems). Relative mRNA levels were determined using the ΔΔCt method. Values were expressed relative to *GAPDH* levels.

### 2.10 Cell Isolation and Culture

Fibroblasts were isolated from C57BL/6 mice colon mucosa or from human colon biopsies (30). Colon biopsies were collected according to standard procedures. This study was approved by the Ethics Committee of Besançon (#14/456; date of approval: July 13^th^, 2021), and all participants signed an informed consent form. Mucosal sheets were treated with 5 mM of EDTA (Life Technologies) in calcium-free HBSS (Life Technologies) five times at 37°C for 15 min to remove the epithelial layer. The mucosal sheets without epithelial cells were then cut into small pieces and cultured in completed RPMI-1640 medium (Life Technologies) containing 10% of FBS (Life technologies), 1% of penicillin/streptomycin and 10 mg/mL of collagenase D (Roche) for 60 min at 37°C, 5% CO_2_. Isolated fibroblasts were cultured in RPMI-1640 medium containing 10% of FBS, 1% of penicillin/streptomycin, 10 mM of sodium pyruvate (Lonza), 1 mM of non-essential amino acids (Lonza) and 0.05 mM of 2 beta-mercaptoethanol (Sigma Aldrich). The cultured fibroblasts were used for experiments after two to four passages. The immortalized MODE-K cell line derived from mouse small IEC was obtained from INSERM U1111 (31). Cells were cultured in completed RPMI-1640 medium at 37°C, 5% CO_2_.

### 2.11 Functional Assays

#### 2.11.1 Proliferation and Phagocytosis

Cell proliferation was determined by mitochondrial activity using the 3-(4,5-dimethylthiazol-2yl)-2,5-diphenyltetrazolium bromide (MTT) assay according to the manufacturer’s instructions. For the phagocytic assay, apoptotic cells were stained with carboxyfluorescein succinimidyl ester (CFSE; Invitrogen) according to the manufacturer’s protocol, and co-cultured with MODE-K cells for six hours in 24-well plates with 1 mL of SuperMApo or X-vivo control medium. Phagocytosis was determined by FACS.

#### 2.11.2 Wound Healing Scratch Assay and Migration Assay

For the wound-healing scratch assay, cells were seeded in 6-well plates (5.10e5 cells/well) and grown to confluence. Then, linear mechanical scratch wounds were performed in each well using a 1000-µl plastic pipette tip, and the rate of cell migration in the presence of 5 mL of SuperMApo or X-vivo control medium was measured by determining the surface empty of cells immediately after wounding (day 0) and at different times as indicated. For migration assays, 8-µm pore size transwells (Greiner) were used with cells seeded on the top of the transwells, and incubated for 24 hrs at 37°C, 5% CO_2_, in the presence of 1 mL of SuperMApo or X-vivo control medium. Then, after removing cells from the top using a cotton bud, cells at the bottom of the wells were fixed with 100% of ethanol, stained with 2% of crystal violet and pictures from each membrane were taken. Finally, the invasive cells located in the lower chamber were counted manually.

#### 2.11.3 Contraction Assay

Concerning the contraction assays, collagen culture gel was prepared as previously described (32). Briefly, collagen was prepared by mixing 0.1% of acetic acid with rat-tail type 1 collagen (Corning®) to 3 mg/mL. Then, 400 µL of cell suspension was mixed with 200 µL of collagen solution in a 24-well plate. Quickly, 1 M of NaOH was added to the well for gel solidification. After 20 min, the gel was finally detached from the bottom of the well and incubated during 24 hrs at 37°C, 5% CO_2_ in the presence of 1 mL of SuperMApo or X-vivo control medium. Gel contractions were determined by the measurement of collagen disk diameters at different time points.

### 2.12 Statistical Analysis

Data are expressed as mean ± standard error of the mean (s.e.m.), and were analyzed using adapted statistical tests, as indicated in figure legends, using GraphPad Prism software (5.01 version). A *p*-value below 0.05 was considered statistically significant.

## 3 Results

### 3.1 Pro-Resolving Factors Released by Macrophages After Efferocytosis Promote the Resolution of Inflammatory Bowel Diseases

**Figure 1:**
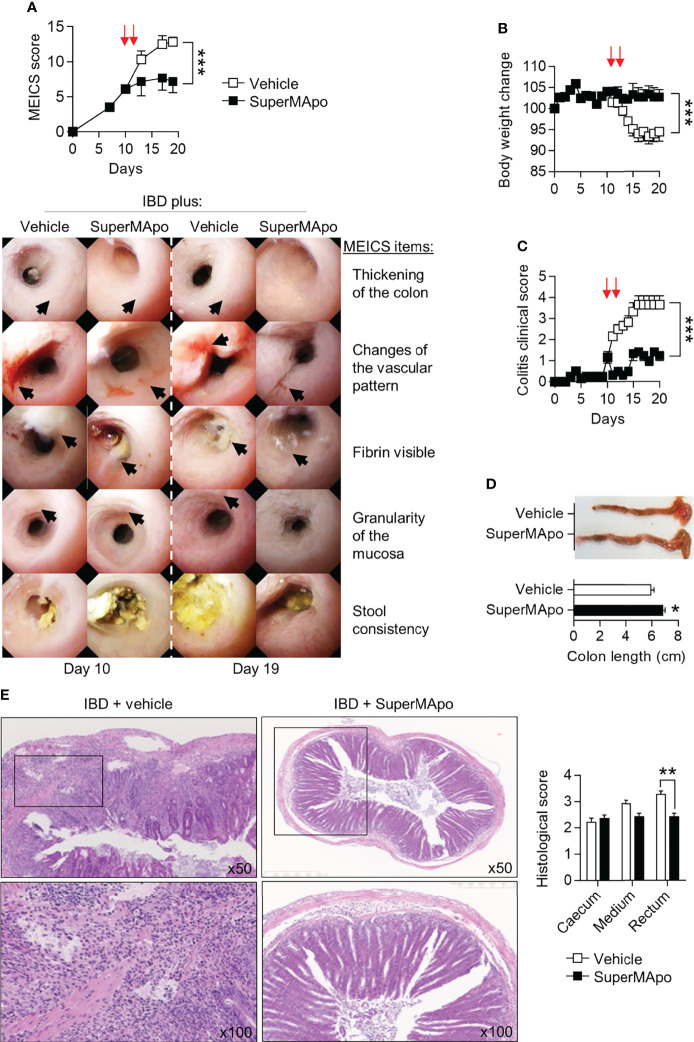
Pro-resolving factors released by macrophages after efferocytosis promote intestine mucosal healing and limit colitis. Colitis was induced by the transfer of naïve CD4^+^CD25^–^CD45RB^hi^ T cells into Rag2^–/–^ C57BL/6 mice. **(A)** Colon examination was performed by colonoscopy and lesion quantification was assessed by the murine endoscopic index of colitis severity (MEICS) score. Mice developing colitis received twice (arrows) pro-resolving factors (SuperMApo) or vehicle (1 mL/mouse, i.p.) 10 days after the induction of colitis (*i.e*., naïve T cell transfer) when they experimented a MEICS score of 6 to 7 out of 15, and 48 h later. Representative pictures are displayed from day 10 and 19 according to MEICS items. Black arrows identify the MEICS items on each picture. Cumulated data from one representative experiment out of three, expressed as mean ± s.e.m. (5 to 6 mice per groups) were also shown. **(B, C)** Body weight loss and clinical score changes were assessed on colitis mice. Data are expressed as mean of group ± s.e.m. (5 to 6 animals per groups) from one representative experiment out of three independent experiments; ***p < 0.001 (two-way ANOVA with Bonferroni post-test). **(D)** Colon length was assessed on colitis mice from a. Representative colon pictures are shown as well as cumulative data from one representative experiment out of three independent experiments (5 to 6 mice per group); *p < 0.05 (nonparametric Mann-Whitney test). **(E)** Representative HES staining of paraffin-embedded sections of the colonic tissues obtained from colitis mice from **(A)**, and cumulative histological scores (pooled from 3 independent experiments) are shown and expressed as mean of group ± s.e.m. (5 to 6 mice per group); **p < 0.01 (nonparametric Mann-Whitney test).

The pro-resolving functions of the factors released by macrophages after co-culture with apoptotic cells (named SuperMApo) have been described previously (14). These factors were evaluated for their capacity to stop naive T cell transfer-induced colitis. SuperMApo was injected twice (at a 48-hr interval) in mice exhibiting ongoing colitis, as attested by a MEICS score of 6 to 7 out of 15 (determined by live colonic endoscopy). The day after the last injection, colonic lesions stopped progressing in SuperMApo-treated mice, as attested by the MEICS score stagnation, whereas these lesions aggravated in vehicle-treated mice (**Figure 1A**). A thickening and increased granularity of the colon mucosa, a high fibrin deposition as well as thinner and tortuous blood vessels demonstrated lesion worsening between the last day of treatment (day 10) and day 19 in vehicle-treated mice (**Figure 1A**). MEICS score improvement after SuperMApo treatment was associated with reduced weight loss and reduced colitis clinical score (**Figures 1B, C**). Intestinal necropsy 10 days post-treatment showed an increased length of the colon in IBD mice receiving SuperMApo compared to the colon length in vehicle-treated mice. Colons of vehicle-treated mice were shorter, hyperemic and contained fewer feces due to massive diarrhea (**Figures 1A, D**). Altogether, this indicates inflamed colons. Histological examination of the intestines showed a reduced infiltration of inflammatory cells, notably in the rectum, in SuperMApo-treated mice (**Figure 1E**). Overall, these data demonstrate that the injection of pro-resolving factors released by efferocytic macrophages in mice with ongoing IBD could control IBD progression and inflammatory cell infiltration.

**Figure 2:**
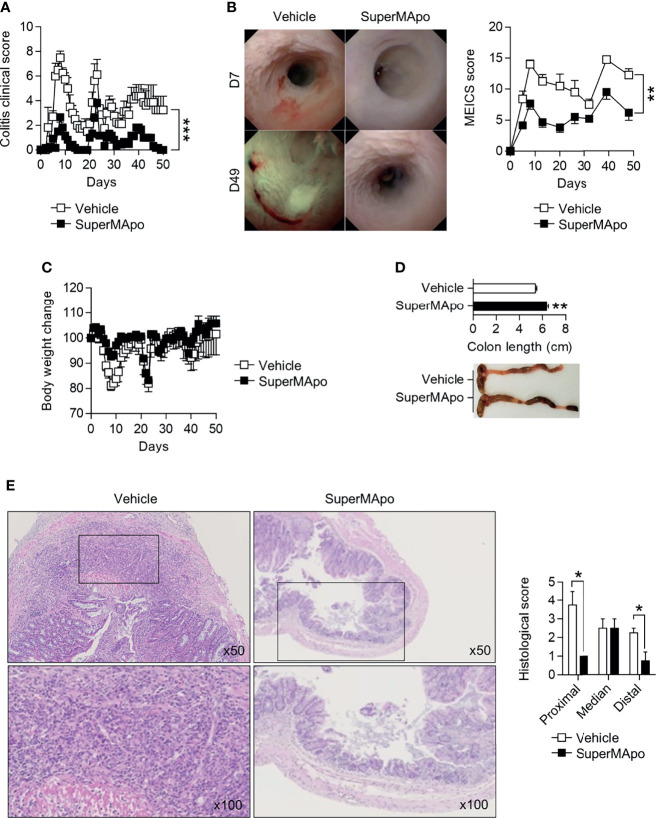
Pro-resolving factors released by macrophages after efferocytosis promote healing of colon lesions induced by dextran sodium sulfate and limit colitis. **(A)** C57BL/6 Mice were subjected to three cycles of DSS and received at day 0 and day 2 pro-resolving factors (SuperMApo, 1 mL/mouse; i.p.) or vehicle, and were monitored daily for clinical changes over 50 days. Data are expressed as mean of group + s.e.m. (5 to 6 mice per group) and are representative of two independent experiments; ***p < 0.001 (two-way ANOVA with Bonferroni post-test). **(B)** Endoscopic assessment according to the MEICS score was also performed on colitis mice from **(A)** as well as body weight loss **(C)**. Representative pictures of colonoscopy at day 7 and day 49 are shown, as well as cumulated MEICS score from two independent experiments. Data are expressed as mean of group + s.e.m. (5 to 6 mice per group); **p < 0.01 (two-way ANOVA with Bonferroni post-test). **(D)** Colon length was assessed on the colitis mice from a, and representative colon pictures were shown as well as cumulative data from two independent experiments. Data are expressed as mean of group + s.e.m. (5 to 6 mice per group); **p < 0.01 (nonparametric Mann-Whitney test). **(E)** Representative HES staining of paraffin-embedded sections of the colonic tissues from colitis mice from a (at x50 and x100 magnifications). Cumulative histological scores from one experiment are shown as mean of group + s.e.m. (5 to 6 mice per group); *p < 0.05 (nonparametric Mann-Whitney test).

We further evaluated the wound healing properties of factors produced by efferocytic macrophages in DSS-induced colitis model. Administered on the day of colitis induction and 48 hrs later, SuperMApo strongly prevented colitis severity as attested by a reduced clinical score (which reached a score of 0 by day 48 following treatment), and a reduced MEICS score (**Figures 2A, B**) throughout the 50 days of the experiments. Body weight loss was not significantly improved (**Figure 2C**). Vehicle-treated mice exhibited worse colitis lesions, such as high fibrin deposition within the colon, a thickened and bleached mucosa with spontaneous bleeding, superficial ulceration and edema (**Figure 2B**). Macroscopic and histologic examinations confirmed mucosa improvement in mice receiving pro-resolving factors (**Figures 2D, E**). Overall, these factors exhibit pro-resolving properties in inflamed intestine mucosa.

### 3.2 Pro-Resolving Factors Released by Macrophages After Efferocytosis Induce Wound Healing Properties in Mouse Fibroblasts and Intestinal Epithelial Cells

**Figure 3:**
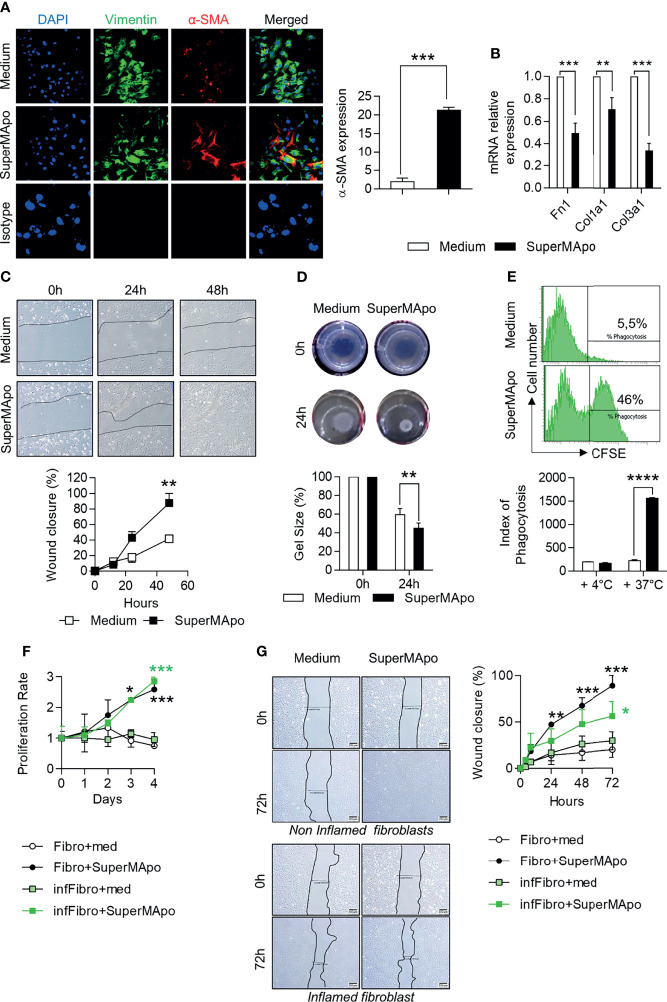
Pro-resolving factors released by macrophages after efferocytosis trigger wound healing properties in fibroblasts and intestinal epithelial cells. **(A)** Expression of vimentin, α-smooth muscle actin (α-SMA) assessed by immunofluorescence and DAPI staining in mouse colon primary fibroblasts cultured in the presence of SuperMApo or control medium for 24 (h) Representative pictures are shown as well as cumulative data representative of α-SMA expression from three independent experiments, as mean ± s.e.m.; ***p < 0.001 (unpaired student’s t test). **(B)** mRNA expression of genes coding for fibronectin (*Fn1*), type I (*Col1a1*) and type III (*Col3a1*) collagen in fibroblasts from **(A)**. Data are expressed as mean of duplicate per condition ± s.e.m. from three independent experiments pooled together. **p < 0.01, ***p < 0.001 (unpaired student’s t test). **(C)** Migration of mouse fibroblasts in wound-healing scratch assays in the presence of SuperMApo or control medium. Data are expressed as mean of duplicates per conditions ± s.e.m. from one representative experiment out of three independent experiments. **p < 0.01 (two-way ANOVA with Bonferroni post-test). **(D)** Contractility of mouse fibroblasts was assessed in floating collagen gels containing medium or SuperMApo. Representative pictures are shown, as well as cumulative data (mean ± s.e.m.) from one representative experiment out of three independent experiments. **p < 0.01 (two-way ANOVA with Bonferroni post-test). **(E)** Engulfment of CFSE-labelled apoptotic cells by the mouse intestinal epithelial cell line MODE-K assessed by flow cytometry after 6 h of co-culture in presence of control medium or SuperMApo. To confirm that this represents an active mechanism and to appreciate CFSE-labelled apoptotic cells stick to MODE-K cells, the same experiment was performed at +4°C instead of 37°C. Data are expressed as representative dot plots showing the percentage of CFSE^+^ MODE-K cells and as index of phagocytosis ± s.e.m. from one representative experiment out of three independent experiments; ****p < 0.0001 (non-parametric Mann-Whitney test). **(F, G)** Primary human fibroblasts harvested from biopsies of non-inflamed (Fibro) or inflamed (infFibro) colon tissue were grown in the presence of medium (med) or SuperMApo. **(F)** Proliferation was determined by MTT assay and **(G)** wound closure was analyzed using the scratch assay at 0, 24, 48 and 72 h of culture. Data are expressed as mean of group ± s.e.m., and are representative of three independent experiments; *p < 0.05, **p < 0.01, ***p < 0.001 (two-way ANOVA with Bonferroni post-test).

Fibroblasts and epithelial cells are the main cells involved in tissue wound healing. Next, we addressed whether these cells are affected by pro-resolving factors released by macrophages after efferocytosis (*i.e*., SuperMApo treatment). First, primary mouse fibroblasts extracted from colon tissues were cultured with SuperMApo. α-SMA expression was strongly increased by SuperMApo and, at the same time, mRNA levels of extracellular matrix-associated genes coding for fibronectin, type I and III collagens, were decreased (**Figures 3A, B**). SuperMApo-treated mouse fibroblasts demonstrated enhanced proliferative and migratory properties (**Supplemental Figures 1A, B**). This was further confirmed using a scratch assay where these fibroblasts showed enhanced wound healing capacities (**Figure 3C**). In addition, using floating collagen gels, SuperMApo-treated fibroblasts demonstrated increased contraction capacities (**Figure 3D**). Thus, *in vitro*, primary fibroblasts isolated from colon demonstrated higher healing properties in the presence of pro-resolving factors released by macrophages after efferocytosis.

We then analyzed the effects on IEC using the MODE-K intestinal immortalized epithelial cell line. We observed a similar improvement of healing functions, such as increased proliferative and migratory capacities, as well as increased wound closure properties (**Supplemental Figures 1D–F**). In addition to the gain in healing properties, SuperMApo allowed MODE-K epithelial cells to acquire phagocytic capacities to eliminate apoptotic cells (**Figure 3E**), as previously demonstrated in the lung mucosa (33). These data indicate that pro-resolving factors produced by efferocytic macrophages trigger wound healing properties in fibroblasts and IEC, providing IEC with phagocytic activity as well.

### 3.3 Pro-Resolving Factors Released by Macrophages After Efferocytosis Restores Healing Properties of Human Fibroblasts Isolated From Patients Diagnosed With IBD

SuperMApo-treated mouse fibroblasts demonstrate all the features of activated fibroblasts but without overexpressing extracellular matrix-associated genes. This suggests that fibroblasts acquired a specific pro-resolving profile. These properties were therefore evaluated on human fibroblasts isolated from colon biopsies (from inflamed lesions and control tissues of patients diagnosed with IBD). In the presence of human SuperMApo (*i.e*., the secretome of human macrophages that have eliminated human apoptotic cells), the wound healing functions of control fibroblasts (*i.e*., isolated from control tissue) were strongly increased including higher proliferative properties and higher wound closure capacities (**Figures 3F, G**). Concerning inflamed lesion-derived fibroblasts, proliferative properties and wound closure capacities were also increased and less modestly (**Figures 3F, G**). These data demonstrate that pro-resolving factors released by efferocytic macrophages could restore the healing functions of IBD fibroblasts, and thus support the use of SuperMApo in IBD patients.

### 3.4 Pro-Resolving Factors Released by Efferocytic Macrophages Limits the Loss of Intestinal Permeability by Inducing Intestinal Epithelial Cell Proliferation and Fibroblast Activation

**Figure 4:**
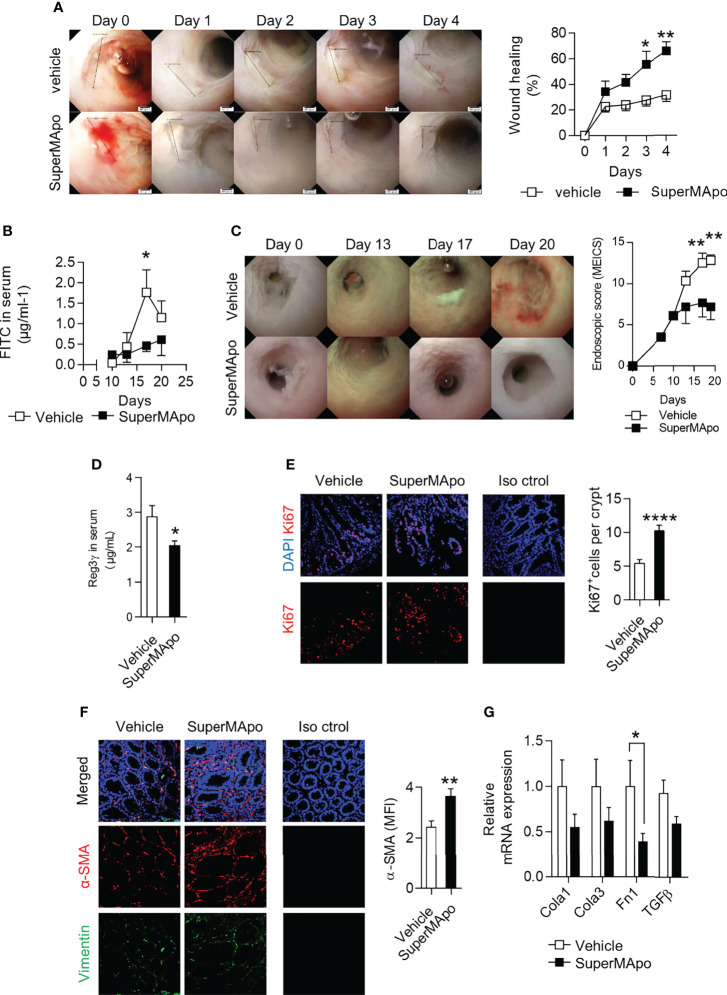
Treatment with pro-resolving factors released by macrophages after efferocytosis maintains intestinal barrier permeability *via* intestinal epithelial cell proliferation and fibroblast activation. **(A)** Wild-type mice were monitored daily by video-endoscopy to evaluate healing of the colon mucosa, wounded at day 0 by forceps and receiving at day 0 and 2 SuperMApo or vehicle (1 mL/mouse; i.p.). Representative pictures are displayed from day 0 to day 4, as well as cumulated data (expressed as % of wound healing, right hand side panel). Data are representative of three independent experiments showing similar results, and expressed as mean of group ± s.e.m. (5 to 6 animals per group); *p < 0.05, **p < 0.01 (two-way analysis of variance [ANOVA] with Bonferroni post-test). **(B–D)** Colitis was induced by transfer of naive CD4^+^CD25^–^CD45RB^hi^ T cells into Rag2^–/–^ C57BL/6 mice and mice received vehicle or SuperMApo at day 10 and 12 (1 mL/mouse; i.p.). **(B)** FITC-dextran particle gavage and quantification in the serum was assessed to appreciate intestinal barrier integrity. Data are expressed as mean of group ± s.e.m. (5 to 6 mice per group) and are representative of two independent experiments; *p < 0.05 (two-way ANOVA with Bonferroni post-test). **(C)** Colonoscopy evaluation of the mice and **(D)** regenerating islet-derived protein 3-gamma (REG3ɣ) quantification in the serum were also performed. Data are expressed as mean of group ± s.e.m. (5 to 6 mice per group) and representative of four independent experiments; **p < 0.01 (two-way ANOVA with Bonferroni post-test). **(E)** Cell proliferation in the epithelial crypts and **(F)** fibroblast differentiation into myofibroblasts were determined by Ki-67 and α-SMA staining, respectively, of colon cuts from colitis mice from **(B)**. Nuclei are counterstained with DAPI and an isotype control antibody was used as control (Iso ctrol). **(G)** Expression of mRNA of genes coding for extracellular matrix proteins type I [*Col1a1*] and type III [*Col3a1*] collagen, fibronectin [*Fn1]*) and TGF-β (*tgf-b*) was assessed by RT-qPCR in the colon of the colitis mice from **(B)** Data are expressed as mean of group ± s.e.m. (5 to 6 mice per group) and are representative of two; *p < 0.05 (Unpaired student t test), **p < 0.01; ****p < 0.0001 (nonparametric Mann-Whitney test).

The enhanced healing functions induced by pro-resolving factors released by efferocytic macrophages were then addressed *in vivo* using a biopsy forceps-wounded colonic mucosa model in which inflammation is localized to the injured tissue and not systemic. In this model, i.p. injection of SuperMApo on the day of mucosa injury significantly accelerated the lesion wound healing observed by video endoscopy, compared to the lesions of control vehicle-treated mice (**Figure 4A**). We addressed the integrity of the intestinal barrier after naive T cell transfer-induced colitis by using FITC-Dextran oral administration and its quantification in the mouse serum. Reduced systemic levels of FITC-Dextran were found in colitis mice receiving SuperMApo compared to control mice (**Figure 4B**). This attested for a conserved barrier permeability, further supported by a lower MEICS score (**Figure 4C**). In addition, the levels of the intestinal barrier integrity marker, the regenerating islet-derived protein 3γ (REG3γ) (34, 35), were also found reduced in colitis mice treated with SuperMApo (**Figure 4D**). These results suggest a restored intestinal barrier permeability by administration of pro-resolving factors. This was associated with a marked *in vivo* proliferation of cells within the crypts and activation of fibroblasts in the colon mucosa of colitis mice receiving SuperMApo, as attested by increased Ki67^+^ cell number per mucosal crypt, and with an increased expression of α-SMA in colonic fibroblasts, respectively (**Figures 4E, F**). Moreover, a slight decrease of colon mRNA expression of extracellular matrix-associated genes *Fn1*, *Col1a1*, *Col3a1* and *Tgf-β* was observed in colitis mice receiving SuperMApo treatment. This decrease was significant for *Fn1* (**Figure 4G**). Overall, our data demonstrate that treatment of mice experiencing ongoing colitis with pro-resolving factors released by efferocytic macrophages restores intestinal barrier permeability by inducing IEC proliferation and activating local fibroblasts *in vivo*.

### 3.5 TGF-β, IGF-I and VEGF Growth Factors Participate in the Wound Healing Induced by Factors Released by Macrophages After Efferocytosis

**Figure 5:**
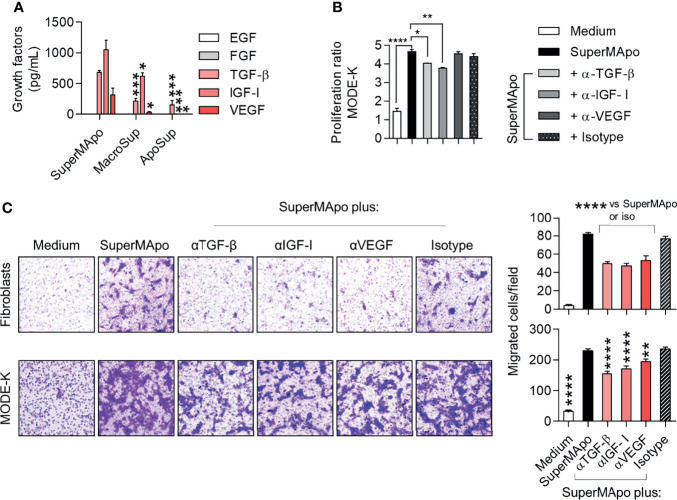
Pro-resolving factors released by macrophages after efferocytosis include TGF-β, IGF-I and VEGF and their capacity to attract primary fibroblasts and IEC are dependent on these growth factors. **(A)** EGF, FGF, TGF-β, IGF-I and VEGF were quantified by ELISA in the supernatants of macrophages after efferocytosis (SuperMApo) and in the control supernatants of macrophages (MacroSup) cultured alone or apoptotic cells (ApoSup) cultured alone. Data represented as mean ± s.e.m. are the pool of six independents experiments; *p < 0.05; ***p < 0.001 (one-way ANOVA with Tukey’s multiple comparison post-test). **(B)** Proliferation of mouse primary fibroblasts was assessed by MTT assay for 4 days with or without neutralizing antibodies to TGF-β, IGF-I or VEGF, or control isotype. Data are from one representative experiment out of three and expressed as mean + s.e.m. of 3 replicates per condition; *p < 0.05; **p < 0.01, ****p < 0.0001 (two-way ANOVA with Bonferroni post-test). **(C)** Mouse primary fibroblasts or MODE-K IEC were incubated with medium or SuperMApo, with or without neutralizing antibodies to TGF-β, IGF-I or VEGF, or control isotype, and migration was determined using a transwell assay during 24 (h). Data are shown as representative pictures and as pooled data expressed as mean of migrated cells/field of duplicates per group ± s.e.m. from one representative experiment out of three. **p < 0.01, ****p < 0.0001 (one-way ANOVA with tukey’s multiple comparisons post-test).

Several pro-resolving factors have been shown to be released by macrophages after efferocytosis (36, 37). Thus, different growth factors, including EGF, FGF, TGF-β, IGF-I and VEGF, were quantified in the SuperMApo supernatant. TGF-β, IGF-I and VEGF were detected in significant higher quantities than in control supernatants, whereas EGF and FGF were not detectable (**Figure 5A**). When blocking antibodies directed against TGF-β, IGF-I and VEGF were used, we observed a non-significant decrease of SuperMApo-induced α-SMA expression (**Supplemental Figure 2A**). The reduction of SuperMApo-induced *Col1a1*, *Col3a1* and *Fn1* mRNA expression in primary fibroblasts was not significantly affected by growth factor blockade (**Supplemental Figure 2B**). The proliferative and contractive properties of mouse primary fibroblasts were not affected by antibodies blocking these growth factors (**Supplemental Figures 2C, D**). However, blockade of TGF-β or IGF-I significantly reduced the proliferation of IEC line MODE-K (**Figure 5B**). In addition, SuperMApo-enhanced cell migration of fibroblasts and IEC was reduced by the addition of blocking antibodies whatever the neutralized growth factor *(i.e*., TGF-β, IGF-I or VEGF) (**Figure 5C**). Thus, while proliferation and migration of IEC induced by SuperMApo are sustained by TGF-β, IGF-I and VEGF growth factors, only migration of fibroblast is associated to these growth factors.

**Figure 6:**
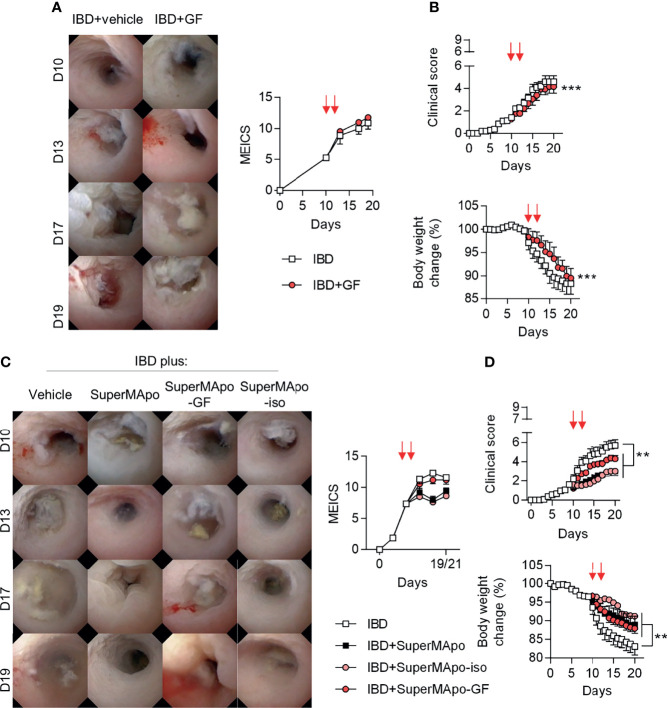
TGF-β, IGF-I and VEGF found in the SuperMApo supernatant participate in lesion healing. **(A, B)** Colitis was induced by the transfer of naive CD4^+^CD25^–^CD45RB^hi^ T cells into Rag2^−/−^ C57BL/6 mice and mice received at day 10 and 12 vehicle (PBS; 1 mL/mouse) or the three following recombinant growth factors: TGF-β (700 pg/mL/mouse), IGF-I (1050 pg/mL/mouse) and VEGF (350 pg/mL/mouse) (red arrows) when they exhibited a MEICS score of 6-7, and were then monitored daily. **(A)** Endoscopic assessment according to the MEICS score was performed and results are shown as representative pictures of mini-colonoscopy at day 10, 13, 17 and 19, as well as cumulated score. **(B)** Clinical score changes and body weight loss were also assessed and results are expressed as mean of groups ± s.e.m. (5 to 6 mice per group). Data are from two independent experiments pooled together; ***p < 0.05, *p* < 0.001 (one-way ANOVA with Bonferroni post-test). **(C)** Colitis was induced similarly and mice received SuperMApo, or SuperMApo depleted in TGF-β, IGF-I and VEGF (SuperMApo-GF), or SuperMApo treated similarly but using isotype control antibodies instead of antibodies against growth factors (SuperMApo-iso), or vehicle when they exhibited a MEICS score of 6-7 and were then monitored daily. Endoscopic assessment according to the MEICS score was performed and results are shown as representative pictures of mini-colonoscopy at day 10, 13, 17 and 19, as well as cumulated score. **(D)** Clinical score changes and body weight loss were also assessed and results are expressed as mean of groups ± s.e.m. (5 to 6 mice per group). Data are from three independent experiments pooled together; **p < 0.01 (one-way ANOVA with Bonferroni post-test).

The role of TGF-β, IGF-I and VEGF growth factors was further addressed *in vivo* using the T cell-transfer colitis model in which all the growth factors tested *in vitro* were infused together. While colon lesions were not ameliorated – as attested by a similar MEICS score between growth factor-treated and control mice (**Figure 6A**), weight loss and the composite clinical score were slightly improved by the treatment with the three growth factors (**Figure 6B**). We next decided to deplete the SuperMApo supernatant from TGF-β, IGF-I and VEGF using antibody-coupled microbeads before its injection to colitis mice. Depletion of TGF-β, IGF-I and VEGF from SuperMApo inhibited the improvement of the MEICS score induced by SuperMApo (**Figure 6C**). However, the absence of TGF-β, IGF-I and VEGF in the SuperMApo supernatant partially reduced the properties of SuperMApo in terms of weight loss prevention and clinical score reduction (**Figure 6D**). These data demonstrate that TGF-β, IGF-I and VEGF participate in intestinal mucosal healing induced by SuperMApo but are not sufficient to resolve global IBD inflammation.

Altogether, these results highlight pro-resolving factors released by efferocytic macrophages as a promising therapeutic approach to both circumvent inflammation and promote lesion healing in the setting of inflammatory bowel diseases.

## 4 Discussion

IBD is a family of inflammatory diseases that ultimately lead to damage of the distal small intestine and the colonic mucosa. Available treatments for IBD based on immunosuppressive agents try to diminish the effector response and treat the sequelae of uncontrolled/nonresolving inflammation. However, these drugs are not entirely effective, exert nonspecific functions and result in multiple adverse effects, including infections. In most cases, surgical resection is the last alternative. This illustrates the need for new therapeutic approaches that specifically modulate both components of the disease, namely uncontrolled inflammation and tissue repair. Recent studies have shown that IBD patients who achieve and maintain colonic mucosal healing have more favorable long-term outcomes than patients who do not. Thus, mucosal healing is emerging as a critical endpoint in clinical trials and practice (2, 7). Resolution pharmacology proposes to trigger the resolution phase of inflammation and initiate tissue repair (38, 39). Apoptotic cell-based therapy can be an interesting approach in this setting, since apoptotic cell infusion has been shown to improve chronic intestinal inflammation in two different experimental colitis models, naive T cell transfer- and DSS-induced colitis (24, 25, 40). These approaches are based on the triggering of the resolution phase of inflammation induced by efferocytosis. Soluble factors released by macrophages after efferocytosis play an important role in the success of apoptotic cell-based therapies (41, 42). Here, the novelty resides in the use of the cell-free whole secreted factors issued from macrophages that have eliminated apoptotic cells in order to reengage and foster the resolution of established inflammation in ongoing colitis. This new biologic complex product SuperMApo, contains all the factors released by efferocytic macrophages. These factors are involved in the resolution of inflammation and in tissue healing (14). In the present work, we wanted to focus on the healing properties of SuperMApo using colitis models in which lesions can be regularly followed up by live endoscopy in order to appreciate mucosa lesion healing (26). How SuperMApo factors affected the cells orchestrating tissue repair, namely fibroblasts and IEC, was addressed in the context of colitis and wound healing. Three *in vivo* models were used in which video-endoscopy (26) allowed us to better appreciate lesion healing. With the naive T cell-transfer model (27), we were able to address the therapeutic effect of SuperMApo injection in mice presenting colon lesions, lesions that are mostly T cell dependent. With the DSS-induced colitis model, the preventing role of SuperMApo treatment (injected at the time of DSS administration initiation) was addressed up to 50 days and, this model also helped us to determine the capacity of the drug candidate to prevent metabolite-induced microbiota alteration favoring mucosal lesion occurrence. Furthermore, using the biopsy forceps-wounded colonic mucosa model (26) we addressed mucosal wound healing in absence of systemic inflammation, focusing on the property of the product to enhance tissue healing. Collectively, the data provide several key insights. First, we demonstrated that SuperMApo stimulates the wound healing properties of colonic fibroblasts and IEC, the main cell types orchestrating the tissue repair process (43–46). *In vitro* treatment of fibroblasts with SuperMApo increases the expression of α-SMA. This is confirmed *in vivo* in naive T cell transfer-induced colitis. This suggests the differentiation of fibroblasts into myofibroblasts, but paradoxically SuperMApo decreases the expression of genes coding for extracellular matrix proteins, particularly *Fn1 in vivo*. Our observation suggests that, under this pro-resolving microenvironment (*i.e*., exposure to SuperMApo), fibroblasts appear as activated (as attested by α-SMA expression) but with reduced pro-fibrotic functions (*i.e*., reduced expression of extracellular matrix genes). Other fibroblast functions involved in wound healing are also stimulated *in vitro* by SuperMApo, including migratory, proliferative and contractive properties. While there is a difference between the biology of mouse and human pro-resolving macrophages (47), human pro-resolving factors (*i.e*., SuperMApo) also increase the capacity of human fibroblasts isolated from colons of IBD patients to proliferate, migrate and close a wound *in vitro*. In addition, this targets human fibroblasts either from inflammatory or non-inflammatory intestinal tissues. Concerning IEC, we also observed a gain of pro-healing properties both *in vitro* and *in vivo* after the reintroduction of pro-resolving factors released by efferocytic macrophages, as well as the acquisition of efferocytic capacities. It has been previously demonstrated that a dysregulated clearance of apoptotic cells is associated with many inflammatory diseases (48, 49), and that boosting apoptotic cell clearance dampens inflammation, notably through increasing colonic epithelial cell efferocytosis of apoptotic cells (33). Therefore, an interesting extrapolation of this work is that improving apoptotic cell clearance by IEC in the early stages of IBD could potentially help to reduce the inflammatory responses seen in later stages of the disease.

Second, pro-resolving factors released by efferocytic macrophages promote mucosal healing *in vivo* in three different intestinal models whatever the initial injury (mechanical [biopsy forceps], T cell-mediated, or chemical [DSS]). SuperMApo stimulates wound healing *via* the pro-healing properties of fibroblasts and IEC. We did not study whether efferocytosis factors also promoted extra-intestinal bone marrow-derived cell recruitment to participate in wound healing, as observed in a recent study using a parabiosis chemical-induced colitis model (50). It has been shown that colonic fibroblasts are critically involved in tissue repair in the gastrointestinal tract (43). In the colon, based on their location (just beneath the IEC) and on their net-like structures, intestinal fibroblasts seem to be ideally located for sensing damage signals resulting from disintegration of the IEC layer (44). Following injury, adjacent IEC rapidly migrate into the damaged area to restore barrier integrity (a process called epithelial restitution). Proliferation of IEC then increases the number of enterocytes to reduce this damaged area resulting from the wound. Furthermore, differentiation of IEC is needed to restore the mucosal barrier and epithelial function (45, 51). Thus, our data confirm that pro-resolving factors contained in SuperMApo initiate these mechanisms involved in intestinal mucosal healing, including migration and activation of fibroblasts into myofibroblasts, which facilitate the wound gap closure, as well as migration and proliferation of IEC. As discussed above, a decrease in extracellular matrix gene expression in the colons of SuperMApo-treated mice has been also observed, despite a higher expression of α-SMA expression in fibroblasts. This is associated with a decrease of *tgf-b* mRNA expression in the colons of SuperMApo-treated mice. TGF-β is produced by a variety of cell types. Macrophages, as the critical source of this growth factor, have been shown to promote fibrosis (52). Thus, TGF-β is considered as fibrotic, as it contributes to myofibroblast differentiation by stimulating α-SMA expression (53) and their survival (54). It also stimulates the production of extracellular matrix proteins by myofibroblasts (54), as well as migration (55) and contraction (56) of myofibroblasts. Our data suggest that within SuperMApo, TGF-β improves myofibroblast contractility to enhance the wound gap closure without promoting extracellular matrix production and possibly fibrosis. Neutralization experiments confirmed that TGF-β in SuperMApo stimulated fibroblast proliferation and migration, as well as IEC migration. Further studies are thus necessary to elucidate whether resolving α-SMA positive “extracellular matrix negative” fibroblasts should be considered as healing myofibroblasts or as advanced healing stage fibroblasts.

Third, the different phases of wound healing are controlled by several proteins, such as chemokines, defensins, growth factors (TGF-β, EGF, FGF, or IGF-I), cytokines (e.g., IL-1β, IL-6 and IL-22), as well as trefoil peptides (57, 58). Here, we focused on 5 but 3 growth factors that were detectable in significant amounts in SuperMApo. This corresponds to TGF-β, IGF-I and VEGF that may participate, at least partially, in SuperMApo-induced wound healing *in vitro* and *in vivo*. While i.p. administration of the corresponding recombinant forms of these growth factors, at concentrations similar to those found in SuperMApo, did not ameliorate mucosal lesions, body weight loss was significantly delayed with recombinant growth factor injection. Depletion of the growth factors from SuperMApo confirm that these 3 factors exert a significant effect on wound healing. Body weight loss and clinical score amelioration were only partially affected by the depletion of these growth factors from SuperMApo. This suggests that other factors present in SuperMApo in combination with growth factors, participate in the global resolution of ongoing colitis. This is in line with what we observed *in vitro*. Growth factors induced fibroblast and IEC migration and, only TGF-β and IGF-I induced the proliferation of MODE-K IEC line. Again, this suggests that other factors in SuperMApo may participate. Whether other SuperMApo factors are directly or indirectly involved in wound healing and participate to the treatment of colitis remains to be determined.

In summary, this work demonstrates that SuperMApo improves ongoing IBD, by controlling inflammation and enhancing mucosal healing in mice. SuperMApo also targets human fibroblasts isolated from colons of IBD patients. Thus, SuperMApo emerges as a potential “bio-mimetic” drug belonging to resolution pharmacology, able to terminate ongoing IBD and achieve mucosal healing.

## Data Availability Statement

The raw data supporting the conclusions of this article will be made available by the authors, without undue reservation.

## Ethics Statement

Colon biopsies were collected according to standard procedures. This study (*i.e*., colon biopsy collection and *ex vivo* analysis of the effects of SuperMApo) was approved by the Ethics Committee of Besançon (CPP#14/456; date of final approval: July 13th 2021), and all participants signed an informed consent form. The study protocol is conformed to the ethical guidelines of the 1975 Declaration of Helsinki. The patients/participants provided their written informed consent to participate in this study. Experimentation (#02831) was approved by the local ethic committee (#58) and the French Ministry of Higher Education and Research (Ministère de l’Enseignement Supérieur, de la Recherche et l’Innovation) and was conducted in accordance with the European Union’s Directive 2010/63.

## Author Contributions

SP conceived and supervised the study. OM-R designed and performed most of the experiments, analyzed and interpreted the data. TG, AD, FB, CC, and MC contributed to some experiments. SV-D performed the histological analysis and scoring of the tissues. CG participated to endoscopy procedures. OM-R, PS, and SP analyzed the data and wrote the manuscript. All authors discussed the results and the manuscript and approved the final manuscript.

## Funding

The work was funded by the Etablissement Français du Sang (2016-02 to S.P.), the Conseil Régional de Franche-Comté (“Soutien au LabEX LipSTIC” 2018/2019 to PS, AAP 2013-2017 to SP and SONY SH800Z cell sorter), and by the Agence Nationale de la Recherche (ANR-11-LABX-0021 to Labex LipSTIC), as well as by the MiMedI project funded by BPI France (grant No. DOS0060162/00), and the European Union through the European Regional Development Fund of the Région Bourgogne-Franche-Comté (grant No. FC0013440). OM-R and CC are supported by a doctoral fellowship from the Ministère de l’Enseignement Supérieur, de la Recherche et de l’Innovation, TG by a doctoral fellowship from the Etablissement Français du Sang and from the Labex LipSTIC.

## Conflict of Interest

FB, MC, PS, and SP are shareholders of MED’INN’Pharma SAS. FB, MC, and SP are employed by MED’INN’Pharma SAS which develops the SuperMApo biologic drug candidate.

The remaining authors declare that the research was conducted in the absence of any commercial or financial relationships that could be construed as a potential conflict of interest.

## Publisher’s Note

All claims expressed in this article are solely those of the authors and do not necessarily represent those of their affiliated organizations, or those of the publisher, the editors and the reviewers. Any product that may be evaluated in this article, or claim that may be made by its manufacturer, is not guaranteed or endorsed by the publisher.
