# Supplementary material for: Pro-Resolving Factors Released by Macrophages After Efferocytosis Promote Mucosal Wound Healing in Inflammatory Bowel Disease
Source: Front Immunol. 2021 Dec 22;12:754475. doi: 10.3389/fimmu.2021.754475 (PMC8727348; doi:10.3389/fimmu.2021.754475)
Supplement: Supplementary file 1 [file DataSheet_1.pdf]

Supplementary Material

Pro-resolving factors released by macrophages after efferocytosis promote mucosal wound healing in inflammatory bowel disease

Omayra Martin-Rodriguez[1], Thierry Gauthier[1], Francis Bonnefoy[1,2], Mélanie Couturier[1,2], Anna Daoui[1], Cécile Chague[1], Séverine Valmary-Degano[3], Claire Gay[4], Philippe Saas[1] and Sylvain Perruche[1,2*]

[1]Univ. Bourgogne Franche-Comté, INSERM, EFS BFC, UMR1098 RIGHT, Interactions Hôte-Greffon-Tumeur/Ingénierie Cellulaire et Génique, Fédération Hospitalo-Universitaire INCREASE, LabEx LipSTIC, F-25000 Besançon, France

[2]MED'INN'Pharma, F-25000 Besançon, France

[3]University Hospital of Besançon, Department of Pathology, F-2500 Besançon, France

[4] University Hospital of Besançon, Department of Gastroenterology, F-2500 Besançon, France

*** Correspondence:** Sylvain PERRUCHE

sylvain.perruche@inserm.fr

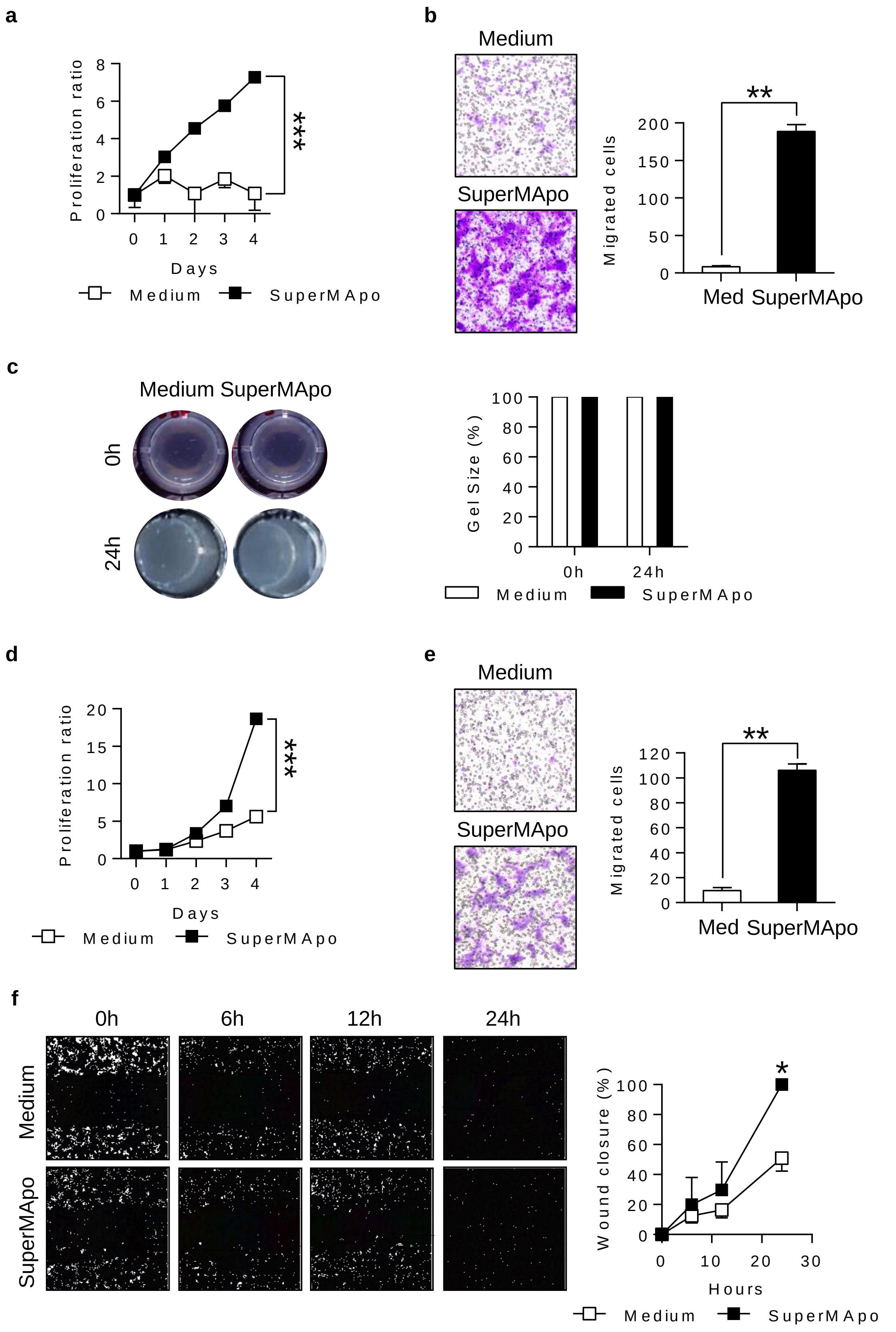

Supplemental Figure 1

Supplementary Figure 1. Pro-resolving factors produced by macrophages after efferocytosis promote pro-repair properties in fibroblasts and IEC. (**a**) MODE-K IEC line was treated with medium or SuperMApo and cell proliferation was monitored by MTT assay for 4 days. Data are expressed as mean ± s.e.m. of 8 replicates per group and are from one representative experiment out of three; ***$p < 0.001$ (two-way ANOVA with Bonferroni post-test). (**b**) Migration of MODE-K cell line treated with medium (Med) or SuperMApo was determined using a transwell assay during 24 h as well as (**c**) contraction properties in collagen gel disk. Results are expressed as mean of duplicates per group ± s.e.m. and are from one representative experiment out of three; **$p < 0.01$ (nonparametric Mann-Whitney test). (**d**) Proliferation of mouse primary colon fibroblasts was monitored by MTT assay for 4 days. Results are expressed as mean ± s.e.m. of 8 replicates per group and are from one representative experiment out of three; ***$p < 0.001$ (two-way ANOVA with Bonferroni post-test). (**e**) Migration of fibroblasts was determined using a transwell assay during 24 h and the results are expressed as mean ± s.e.m. of duplicates per condition and are from one representative experiment out of three. **$p < 0.01$ (nonparametric Mann-Whitney test). (**f**) Wound-healing properties of MODE-K cells were assessed using a scratch assay at 0, 6, 12 and 24 h of culture in the presence of SuperMApo or medium. Results are shown as representative pictures as well as cumulative data, which are represented as mean ± s.e.m. of conditions from one representative experiment out of 3; *$p < 0.05$ (two-way ANOVA with Bonferroni post-test).

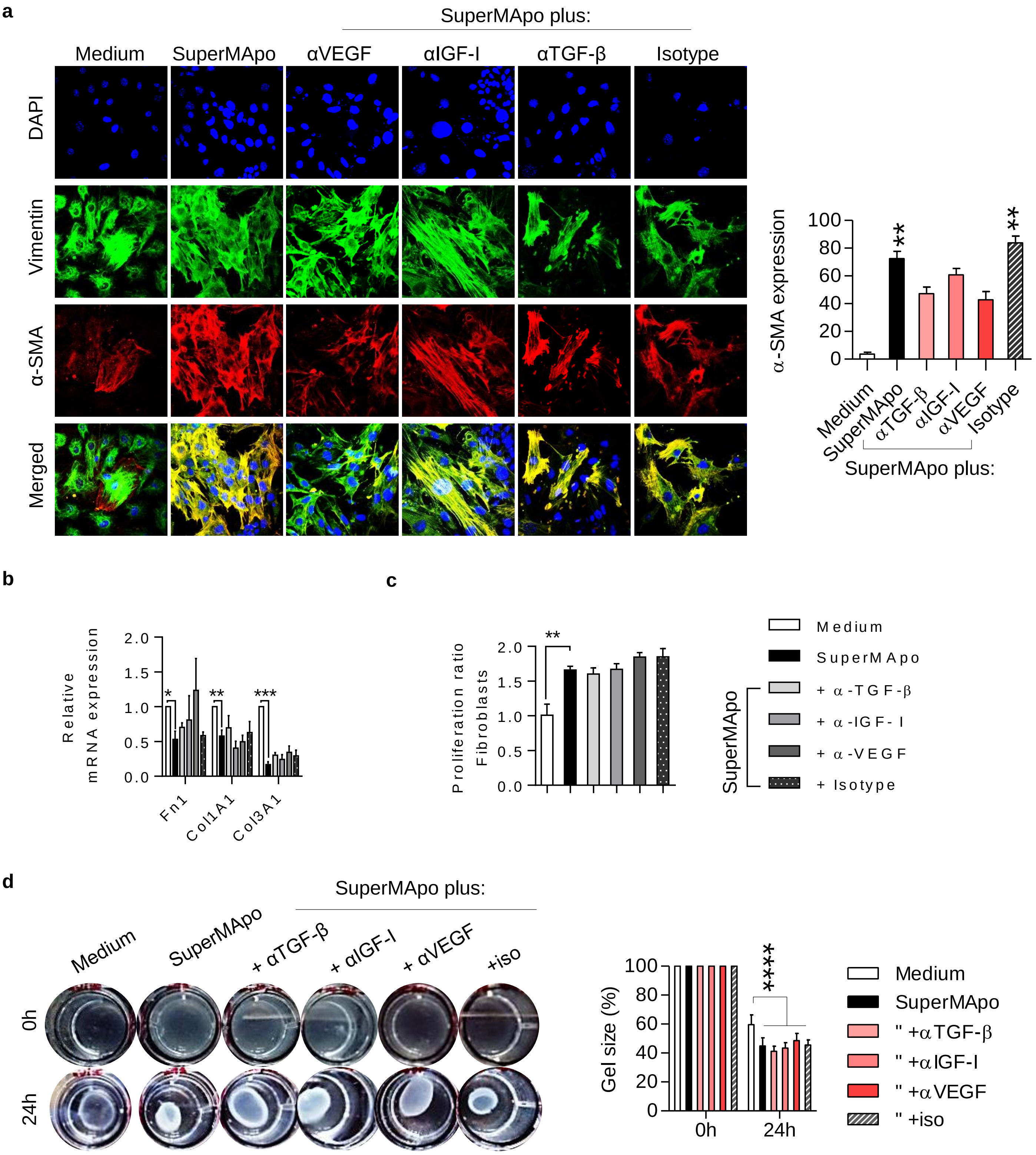

Supplemental Figure 2

Supplementary Figure 2. TGF-β, IGF-I and VEGF –contained within the supernatant SuperMApo– are neither responsible for reducing extracellular matrix gene expression in primary fibroblasts, nor participating in their proliferation nor their contractive properties. (**a**) Mouse colon mucosa primary fibroblasts were cultured in control medium or SuperMApo, with or without neutralizing growth factor antibodies directed against TGF-β, IGF-I or VEGF, or with control isotype, and α-SMA expression was evaluated by confocal microscopy. Vimentin expression is also assessed and nuclei are stained using DAPI. Data are shown as representative pictures and as pooled data expressed as mean of three replicates per group ± s.e.m.; $**p < 0.01$ (vs medium; one-way ANOVA with tukey's multiple comparisons post-test). (**b**) mRNA expression levels of genes coding for fibronectin (*Fn1*), type I (*Col1a1*), and type III (*Col3a1*) collagens were analyzed by RT-qPCR in primary colonic fibroblasts cultured with medium or SuperMApo, with or without neutralizing antibodies directed against TGF-β, IGF-I, or VEGF, or with isotype controls. Data are from three independent experiments and expressed as mean ± s.e.m. of duplicate per condition; $*p < 0.05$, $**p < 0.01$, $***p < 0.001$ (unpaired student's t test). (**c**) Proliferation of mouse primary fibroblasts was assessed by MTT assay for 4 days in the same conditions than in **a**. Data are from one representative experiment out of three and expressed as mean ± s.e.m. of 3 replicates per condition; $*p < 0.05$; $**p < 0.01$, $****p < 0.0001$ (two-way ANOVA with Bonferroni post-test). (**d**) Contraction of mouse primary fibroblasts in floating collagen gels cultured in the same conditions than in **a** was performed and results are shown as representative pictures and cumulative graph from one representative experiment out of three; $****p < 0.01$ (two-way ANOVA with Bonferroni post-test).
